# Hemoadsorption with CytoSorb^®^ and the early course of linezolid plasma concentration during septic shock

**DOI:** 10.1007/s10047-021-01274-4

**Published:** 2021-05-28

**Authors:** Thomas Köhler, Elke Schwier, Carmen Kirchner, Günther Winde, Dietrich Henzler, Claas Eickmeyer

**Affiliations:** 1 Department of Anesthesiology, Surgical Intensive Care, Emergency and Pain Medicine, Ruhr University Bochum, Klinikum Herford, Schwarzenmoorstraße 70, D, 32049 Herford, Germany; 2 Department of General and Visceral Surgery, Thoracic Surgery and Proctology, Ruhr University Bochum, Klinikum Herford, Herford, Germany

**Keywords:** Blood stream infection, Distribution volume, Drug monitoring, Loading dose

## Abstract

Hemoadsorption with CytoSorb^®^ becomes increasingly established in treatment of various, predominantly inflammation-associated diseases. In septic shock, results suggest improvements in hemodynamics and organ function. However, little is known about the in vivo adsorption properties for various antibiotics. We present the case of a 61-year-old female patient with known Ulrich Turner syndrome who treated supportively with CytoSorb^®^ and with linezolid due to a Staphylococcus epidermidis bloodstream infection as part of her intensive care treatment for septic shock. After establishment of a new adsorber, 600 mg of linezolid administered over 1 h. Linezolid levels measured before adsorber inlet (c_pre_) and after adsorber outlet (c_post_) at 0, 15, 60, 120 and 480 min after starting infusion. Out of the ten samples, only the cpre samples 60 min (3.25 mg/l) and 120 min (4.7 mg/l) showed sufficiently high linezolid levels (therapeutic range 3–9 mg/l). After 480 min, cpre decreased to 2.8 mg/l, cpost increased to 1.85 mg/l, and thus clearance decreased to 67.86 ml/min (from 200 ml/min at 60 min), with greatly reduced adsorption capacity of CytoSorb^®^ after 8 h. A loading dose (additional 600 mg) would have been urgently needed. Linezolid therapy under hemadsorption with CytoSorb^®^ requires a clear indication and close monitoring of levels to avoid underdosing.

**Supplementary Information:**

The online version contains supplementary material available at 10.1007/s10047-021-01274-4.

## Background

Hemoadsorption with CytoSorb^®^ (CytoSorbents Medical Inc. Monmouth Junction, New Jersey U.S.A.) is adjunctive therapeutic option for septic shock. The goal is therapeutic control of excessive and dysregulated host response to infection by broad-spectrum adsorption of excessive levels of inflammatory mediators. Results should include hemodynamic stabilization, metabolic improvement, and possibly a reduction in mortality [1]. Other, mostly hydrophobic substances with molecular weights ≤ 50 kDa also adsorbed non-specifically in a size-selective manner, including different antibiotics, e.g. linezolid [2–4].

Linezolid serum levels may be too low in approximately 50% of ICU patients under standard dosing [7]. CytoSorb^®^ may exacerbate this problem, although one case report showed no relevant effect for linezolid [8]. Various adsorption phenomena are known [3], structured clinical studies completely lacking.

## Case presentation

A 61-year-old woman transferred to the intensive care unit postoperatively with stable hemodynamics after right hemicolectomy and extubated within 24 h. Despite administration of cefuroxime and metronidazole, the patient developed septic shock. Antibiosis escalated and vasopressor support restarted at high dosage. Due to acute renal failure, we initiated CVVHD (Multifiltrate, Fresenius Bad Homburg, Germany) with CytoSorb^®^ under citrate anticoagulation to control the cytokine storm, stabilize hemodynamics and establish liver support. CytoSorb^®^ therapy was administered over 94 h. Blood flow (BF) was 200 ml/min, with an amount of purified blood (ABP = therapy time [min] × BF [ml/min]/kg body weight) of 11.05 l/kg. Five adsorbers were used (average 18.8 h/adsorber).

Under adjunctive hemoadsorption with high ABP, the clinical situation stabilized. Norepinephrine support decreased (−60%). Hyperinflammation rapidly controlled (IL6 −93%). Massively elevated static liver function parameters decreased by 95% (ASAT) and 74% (ALAT) under CytoSorb^®^. Meanwhile, serum bilirubin concentration, a marker of intrahepatic bile duct integrity, remained normal. Hemoadsorption was stopped after clinical stabilization (Table 1).

**Table 1:** Important laboratory parameters during CytoSorb^®^ hemoadsorption therapy

Time (h)	Norepinephrine (µg/kg/min)	Leucocytes (Gpt/l)	Interleukin-6 (pg/ml)	ASAT (U/l)	ALAT (U/l)	Bilirubin (mg/dl)	Adsorber number
−4	0.65	21	7.1	31	17	0.13	
20	0.33	35	464	6975	1715	0.46	1
44	0.2	26.6	100	3737	1172	0.47	2
68	0.26	34	61	921	753	0.44	3
92	0.26	44.2	32.5	361	448	0.6	5

Time (h) refers to the beginning of hemoadsorption therapy. CytoSorb was discontinued after 92 h

Detection of *Staphylococcus epidermidis* in multiple blood cultures required additional linezolid. A new adsorber was connected at the start of the infusion (600 mg/300 ml/60 min). For linezolid drug monitoring under CVVHD and hemadsorption, pre- and postadsorber samples were collected 0, 15, 60, 120 and 480 min after the start of infusion.

**Fig. 1:**
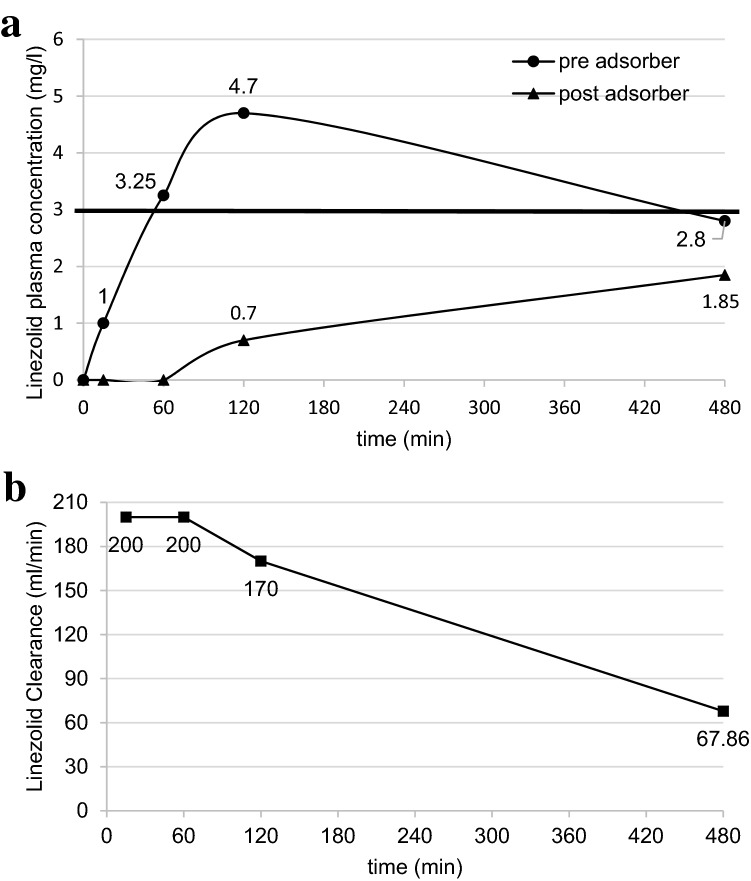
**a** Linezolid plasma concentrations pre- and post-adsorber over the first 480 min after linezolid infusion start. Total infusion time 60 min. Linezolid therapeutic range 3–9 mg/l. **b** Linezolid–Clearance (ml/min) over time

The c_pre_ (systemic levels) were in the therapeutic range (3–9 mg/l) after 60 and 120 min. Clearance decreased already after 60 min (Fig. 1).

**Fig. 2:**
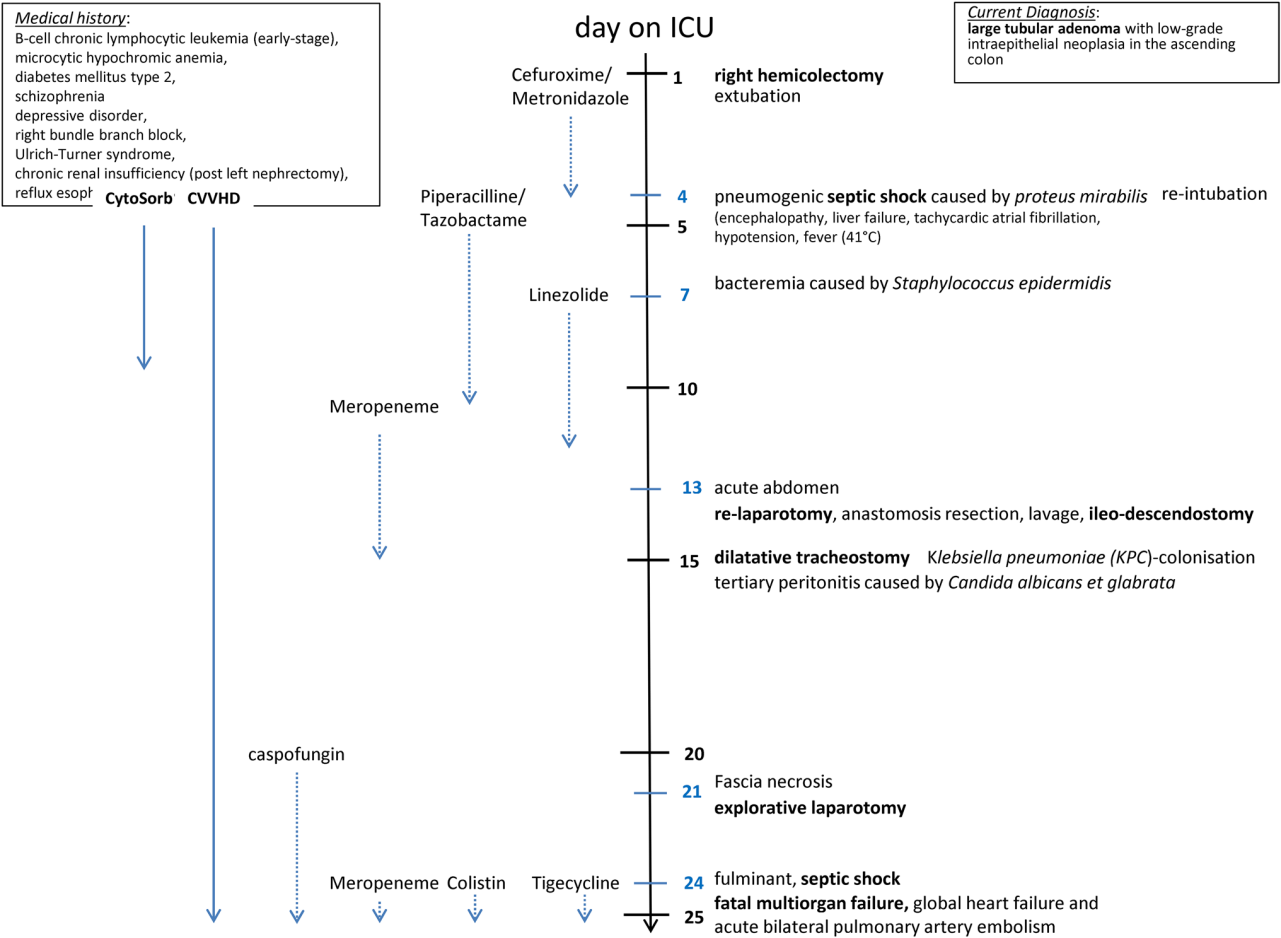
Course of ICU stay with important anamnestic, therapeutic and operative information

Five days later, during a re-laparotomy, the patient found to have a covered insufficiency of the ileo-transversostomy. After a complicated course, the patient succumbed to septic multiorgan failure in global heart failure associated with acute bilateral pulmonary artery embolism and known Ulrich–Turner syndrome (Fig. 2).

## Discussion and conclusions

CytoSorb^®^ extracts various antimicrobial substances at high rates in vitro [3]. Accurate in vivo pharmacokinetic data for antimicrobials during hemoadsorption are scare. Adsorption clearance is highest within the first two hours, making dose adjustment difficult to extrapolate [2].

Linezolid (oxazolidinone) inhibits bacterial protein biosynthesis of Gram-positive bacteria including MRSA and VRE.

The key pharmacological data can be found in Table 1 e [5, 7].

LC–MS/MS was used as linezolid-measurement method. In contrast to previous HPLC methods, interference of the measurement results with the diverse intensive care medication is very unlikely [7].

Antimicrobial chemotherapy should start within the first hour after sepsis diagnosis ("1-h bundle"). Dose-influencing factors include volume of distribution, circulatory status, hepatic and renal function. Therefore, antibiotic dosing is difficult. Continuous renal replacement therapy (CRRT) further alters antibiotic pharmacokinetics and increases the risk of over- or underdosing [6].

Zoller et al. measured linezolid concentrations in the arterial blood of a patient, treated with CytoSorb^®^ and linezolid (1st day 4 × 600 mg, then 2 × 600 mg daily,) on average every 205 min. Levels were within the therapeutic range for 4 days [8]. However, plasma levels not consistently measured in relation to the four hemoadsorption treatments. Only 8 of 25 samples collected simultaneously, during ongoing hemoadsorption. Linezolid adsorption under continuous CytoSorb^®^ therapy remained unclear.

To detect possible linezolid underdosing, we closely monitored the concentrations for the first linezolid dose before and after the adsorber. The fourth unsaturated cartridge installed simultaneously with the infusion of the first 600 mg linezolid/1 h. At its end, c_pre_ (≙ of serum concentration) was 3.25 mg/l (therapeutic range 3–9 mg/l). c_pre_ increased moderately after 120 min and was again subtherapeutic after 480 min. In this context, the time-dependent antibacterial efficacy must take into account [5].

According to the Nadler formula, the blood volume here is 4759 ml. Of this, 200 ml/min (12 l/h) passed over the adsorber and linezolid is first completely removed. The total blood volume formally contacts the adsorber almost 3 times/h. The linezolid concentration continues to increase until the end of the infusion after 60 min (3,25 mg/l), but not to the expected extent.

However, based on the volume of distribution (*V*_d_) of 50 l and the standard dose of 600 mg [5], a linezolid-concentration of 12 mg/l after 60 min would be expected:

$$ {\text{concentration}}\left[ {{\text{mg}}/{\text{l}}} \right] = {\text{dose}}\left[ {{\text{mg}}} \right]/V_{{\text{d}}} . $$

Thus, the therapeutically required rapid increase in concentration (c_pre_) is significantly slowed down under CytoSorb^®^ therapy. A dose escalation would have been urgently required. To estimate the necessary dose, the determination of the *V*_d_ is helpful.

Assuming that linezolid is administered as a rapid injection exclusively into the central compartment, the continuous processes of redistribution, binding and elimination can be neglected (1-compartment model).

After 60 min, the *V*_d_ is *184.6 l* (V_d_ = 600 mg/3.25 mg/l), giving a clear indication of a high, initial CytoSorb^®^ adsorption rate, compared to the known *V*_d_ (50 l). If a "target" concentration of 6 mg/l is set and the calculated *V*_d_ (184.6 l) is used as a basis, this results in a linezolid dose of 1107 mg. This would have required an additional administration of approx. 600 mg linezolid (loading dose).

c_post_ never formally reached sufficient levels (Fig. 1a), i.e. the antibiotic is effectively removed by the adsorber. The new, unsaturated CytoSorb^®^ even completely removed linezolid from the blood within the first hour (c_post_: < 0.5 mg/l). I.e. the maximum adsorption capacity of the CytoSorb^®^ cartridge was not reached during this period, despite a BF of 200 ml/min.

Only thereafter did c_post_ increase measurably (Fig. 1a). With regard to the effective antibiotic level, cpost, in contrast to clearance, is of little significance on its own. Clinically significant influencing factors (altered distribution volumes, altered plasma protein binding, altered albumin concentration, etc.) are of little relevance to the actual adsorption.

Linezolid clearance (ml/min) is calculated as follows:

Clearance (ml/min):=BF × ([c_pre_] − [c_post_] / [c_pre_].

Previous studies [2] and our measurements proved that the CytoSorb^®^ clearance as a measure for the adsorption capacity decreases in a time-dependent manner (Fig. 1b). Especially after adsorber changes, subtherapeutic linezolid levels must expected for a longer time. The dialyzability of linezolid further complicates this. Thus, in addition to "loss" by adsorption, elimination must also considered.

Linezolid adsorption can not always be accurately predicted as a time-dependent function, because clearance depends proportionally on BF. The clearance-curve may be steeper or flatter.

Although the linezolid infusion ended, cpre continued to increase until 120 min. *V*_d_ at this time point was theoretically 127.65 l. Several explanations are possible for this. First, CytoSorb^®^ clearance decreased. Second, redistribution processes from slow compartments can not excluded due to the high *V*_d_. Third, sepsis-related capillary leakage complicates pharmacokinetic considerations immensely. Fourth, it is possible that the linezolid infusion wasn`t terminated after exactly 60 min.

We report a relevant "anti-antibiotic effect" with linezolid underdosing under CytoSorb^®^ therapy. In addition to close therapeutic drug monitoring (TDM), an additional loading dose of 600 mg would have been required at the start of therapy. Other dosages, e.g. 2 × 900 mg or 3 × 600 mg linezolid, seem equally possible. Additional linezolid doses must be correlated with the changing interval due to the initial high CytoSorb^®^ adsorption capacity. Known hepatotoxicity should be addressed and dynamic liver monitoring followed by dose adjustment should be considered [9].

According to drug guidance, 2 × 600 mg of linezolid should always be administered for any age, weight, and indication. It remains to be discussed, whether the "one size fits all" dosing for linezolid is correct under CytoSorb^®^ therapy.

The adsorption curve shown for linezolid may provide important clues to the saturation kinetics of CytoSorb^®^ for other antimicrobials (3). Maximizing blood flow to increase "purified blood volume" for sepsis control may result in higher clearance of antimicrobials, shown here for linezolid, in the first few hours of treatment. Similarly, an increase in clearance rate may be caused by a shorter CytoSorb^®^ change interval. As an intensivist, consider possible dose adjustment for antimicrobials under new-onset hemoadsorption with CytoSorb® or after cartridge change.

Structured, prospective in vivo studies should be conducted in the near future investigating changes in linezolid concentrations under hemoadsorption in short intervals. In addition, close attention should paid to a potentially required dose adjustment.

## Supplementary Information

Below is the link to the electronic supplementary material.

Supplementary file1 (PDF 53 KB)

## Abbreviations

ABP: Amount of blood purified [ml/kg]
ALAT: Alanine aminotransferase
ASAT: Aspartate aminotransferase
BF: Blood flow rate [ml/min]
Cpre: Linezolid levels measured before adsorber inlet
Cpost: Linezolid levels measured after adsorber outlet
CVVHD: Continous veno-venous hemodialysis
HPLC: High performance liquid chromatography
ICU: Intensive care unit
IL-6: Interleukin-6
LC–MS/MS: Tandem mass spectrometry
MIC: Minimum inhibitory concentration
MRSA: Methicillin resistant *Staphylococcus aureus*
PPB: Plasma protein binding rate
CRRT: Continuous renal replacement therapy
VRE: Vancomycin resistant *Enterococcus faecium*
V_d_: Distribution volume

## References

[1] Brouwer WP, Duran S, Kuijper M, Ince C (2019). Hemoadsorption with CytoSorb shows a decreased observed versus expected 28-day all-cause mortality in ICU patients with septic shock: a propensity-score-weighted retrospective study. Crit Care.

[2] König C, Röhr AC, Frey OR, Brinkmann A, Roberts JA, Wichmann D (2019). In vitro removal of anti-infective agents by a novel cytokine adsorbent system. Int J Artif Organs.

[3] Köhler T, Pletz MW, Altmann S, Kirchner C, Schwier E, Henzler D (2021). Pericarditis caused by *Enterococcus faecium* with acute liver failure treated by a multifaceted approach including antimicrobials and hemoadsorption. Case Rep Crit Care.

[4] Poli EC, Rimmelé T, Schneider AG (2019). Hemoadsorption with CytoSorb. Intensive Care Med.

[5] Hashemian SMR, Farhadi T, Ganjparvar M (2018). Linezolid: a review of its properties, function, and use in critical care. Drug Des Devel Ther.

[6] Roberts DM, Liu X, Roberts JA, Nair P, Cole L, Roberts MS (2015). A multicenter study on the effect of continuous hemodiafiltration intensity on antibiotic pharmacokinetics. Crit Care.

[7] Zoller M, Maier B, Hornuss C, Neugebauer C, Döbbeler G, Nagel D (2014). Variability of linezolid concentrations after standard dosing in critically ill patients: a prospective observational study. Crit Care.

[8] Zoller M, Döbbeler G, Maier B, Vogeser M, Frey L, Zander J (2015). Can cytokine adsorber treatment affect antibiotic concentrations? A case report. J Antimicrob Chemother.

[9] Kirchner C, Sibai J, Schwier E, Henzler D, Eickmeyer C, Winde G (2019). Dosing of antimycotic treatment in sepsis-induced liver dysfunction by functional liver testing with LiMAx^®^. Case Rep Crit Care.

