# Supplementary material for: Hemoadsorption with CytoSorb® and the early course of linezolid plasma concentration during septic shock
Source: J Artif Organs. 2021 May 28;25(1):86–90. doi: 10.1007/s10047-021-01274-4 (PMC8866295; doi:10.1007/s10047-021-01274-4)
Supplement: Supplementary file 1 — Supplementary file1 (PDF 53 KB) [file 10047_2021_1274_MOESM1_ESM.pdf]

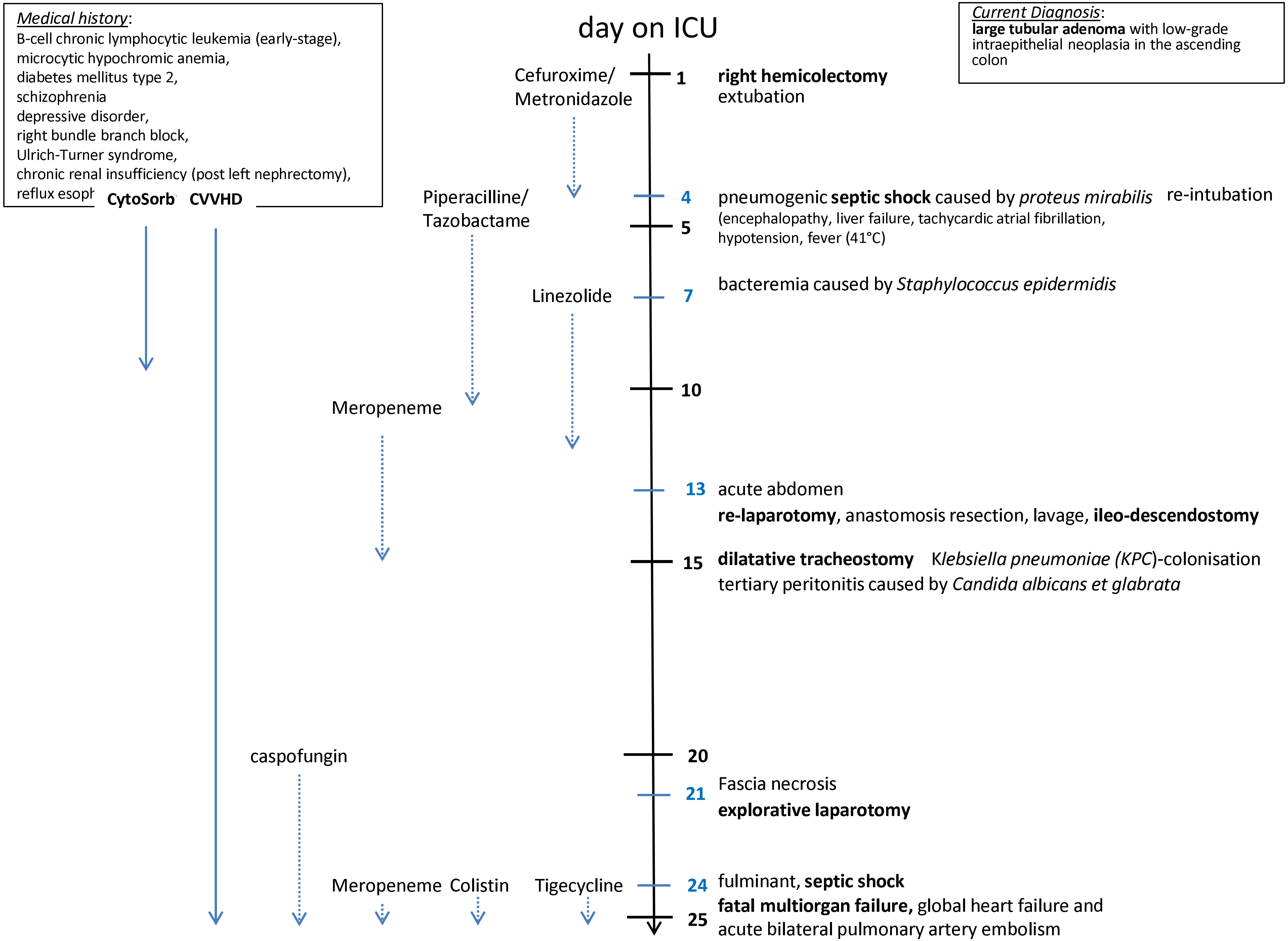
Medical history:
B-cell chronic lymphocytic leukemia (early-stage),
microcytic hypochromic anemia,
diabetes mellitus type 2,
schizophrenia
depressive disorder,
right bundle branch block,
Ulrich-Turner syndrome,
chronic renal insufficiency (post left nephrectomy),
reflux esoph
CytoSorb
CVVHD
day on ICU
Current Diagnosis:
large tubular adenoma with low-grade intraepithelial neoplasia in the ascending colon
Cefuroxime/ Metronidazole
1
right hemicolectomy
extubation
Piperacilline/ Tazobactame
4
pneumogenic septic shock caused by proteus mirabilis
re-intubation
5
(encephalopathy, liver failure, tachycardic atrial fibrillation, hypotension, fever (41°C)
Linezolide
7
bacteremia caused by Staphylococcus epidermidis
10
Meropeneme
13
acute abdomen
re-laparotomy, anastomosis resection, lavage, ileo-descendostomy
15
dilatative tracheostomy
Klebsiella pneumoniae (KPC)-colonisation
tertiary peritonitis caused by Candida albicans et glabrata
caspofungin
20
21
Fascia necrosis
explorative laparotomy
Meropeneme
Colistin
Tigecycline
24
fulminant, septic shock
25
fatal multiorgan failure, global heart failure and acute bilateral pulmonary artery embolism
